# Expression of Cannabinoid Receptors in Human Osteoarthritic Cartilage: Implications for Future Therapies

**DOI:** 10.1089/can.2015.0001

**Published:** 2016-01-01

**Authors:** Sara L. Dunn, Jeremy Mark Wilkinson, Aileen Crawford, Rowena A.D. Bunning, Christine L. Le Maitre

**Affiliations:** ^1^ Faculty of Health and Wellbeing, Biomolecular Sciences Research Centre, Sheffield Hallam University, Sheffield, United Kingdom.; ^2^ Academic Unit of Bone Metabolism, Department of Human Metabolism, University of Sheffield, Sheffield, United Kingdom.; ^3^ Centre for Biomaterials and Tissue Engineering, School of Clinical Dentistry, University of Sheffield, Sheffield, United Kingdom.

**Keywords:** articular cartilage, cannabinoid receptors, cannabinoids, osteoarthritis

## Abstract

**Introduction:** Cannabinoids have shown to reduce joint damage in animal models of arthritis and reduce matrix metalloproteinase expression in primary human osteoarthritic (OA) chondrocytes. The actions of cannabinoids are mediated by a number of receptors, including cannabinoid receptors 1 and 2 (CB1 and CB2), G-protein-coupled receptors 55 and 18 (GPR55 and GPR18), transient receptor potential vanilloid-1 (TRPV1), and peroxisome proliferator-activated receptors alpha and gamma (PPARα and PPARγ). However, to date very few studies have investigated the expression and localization of these receptors in human chondrocytes, and expression during degeneration, and thus their potential in clinical applications is unknown.

**Methods:** Human articular cartilage from patients with symptomatic OA was graded histologically and the expression and localization of cannabinoid receptors within OA cartilage and underlying bone were determined immunohistochemically. Expression levels across regions of cartilage and changes with degeneration were investigated.

**Results:** Expression of all the cannabinoid receptors investigated was observed with no change with grade of degeneration seen in the expression of CB1, CB2, GPR55, PPARα, and PPARγ. Conversely, the number of chondrocytes within the deep zone of cartilage displaying immunopositivity for GPR18 and TRPV1 was significantly decreased in degenerate cartilage. Receptor expression was higher in chondrocytes than in osteocytes in the underlying bone.

**Conclusions:** Chondrocytes from OA joints were shown to express a wide range of cannabinoid receptors even in degenerate tissues, demonstrating that these cells could respond to cannabinoids. Cannabinoids designed to bind to receptors inhibiting the catabolic and pain pathways within the arthritic joint, while avoiding psychoactive effects, could provide potential arthritis therapies.

## Introduction

A key feature of osteoarthritis (OA) is the loss of articular cartilage.^1^ Cartilage breakdown is mediated by complex interactions of proinflammatory cytokines such as interleukin 1 (IL-1) and proteases, including matrix metalloproteinases (MMPs).^2^ This results in a shift in the equilibrium between anabolic and catabolic processes that maintain the extracellular matrix of predominantly collagen and proteoglycan under normal physiological conditions, resulting in cartilage breakdown.

Cannabinoids were originally derived from the cannabis plant, *Cannabis sativa*, which has been used medicinally and recreationally for many years because of its anti-inflammatory, analgesic, and psychoactive properties.^3^ They also include synthetic cannabinoids, such as WIN-55, 212-2 mesylate (WIN-55), and endogenous cannabinoids, (the endocannabinoids), such as anandamide (arachidonoylethanolamide [AEA]). Cannabinoids have been studied in animal models of arthritis and have been shown to reduce joint damage and have anti-inflammatory effects. Evidence suggests that activation of cannabinoid receptors may be of therapeutic value in the treatment of arthritis,^4–8^ and we have previously shown that synthetic cannabinoid WIN-55 reduces the expression of MMP-3 and −13 in OA chondrocytes.^9^

Cannabinoids produce their effects by binding to and activating cannabinoid receptors.^10^ Cannabinoid receptor 1 (CB1) and cannabinoid receptor 2 (CB2) were originally identified as the first two classical cannabinoid receptors.^11,12^ It is now apparent that not all cannabinoid actions are mediated by CB1 and CB2.^10^ Other receptors including peroxisome proliferator-activated receptors alpha and gamma (PPARα and PPARγ), G-protein-coupled receptor 55 (GPR55), G-protein-coupled receptor 18 (GPR18), and transient receptor potential vanilloid 1 (TRPV1) have been identified as cannabinoid receptors.^13–18^

The cannabinoid system has been identified in different cell types of the joint involved in OA and rheumatoid arthritis (RA), including bone cells, synovial cells, and chondrocytes.^19–38^ CB1 and CB2 receptors are expressed in bovine and human chondrocyte cultures, human synovial tissue, and human fibroblast-like synoviocyte cultures.^19,20,30,39^ Moreover, studies using the destabilized medial meniscus (DMM) model of OA have shown that CB2-deficient mice experienced more severe OA than wild-type (WT) OA mice and showed increased susceptibility to age-related OA.^8^ Selective CB2 agonist, HU308, also reduced the severity of OA in WT control mice but not in CB2-deficient mice.^8^ The cannabinoid system in joint cells is, therefore, a potential target in the treatment of arthritis.

The PPAR nuclear receptors family has been shown to bind a number of different cannabinoid ligands and is thought to have anti-inflammatory properties mediated primarily by PPARγ activation.^13^ These receptors are expressed at the mRNA and protein levels in rat growth plate chondrocyte cultures, human cartilage, and isolated chondrocytes.^21–24,26,27^ PPAR activation by both cannabinoid and noncannabinoid ligands displays chondroprotective activities in both OA and RA.^13,21,27,38,40–42^ PPARγ protein expression has been shown to be down regulated in human OA cartilage tissue, suggesting that it may be involved in OA pathogenesis.^22^

PPARγ mRNA expression is down regulated in human OA chondrocytes treated with IL-1, and both PPARγ and PPARα ligands also inhibited IL-1-induced MMP and nitric oxide production in human chondrocytes, suggesting that these receptors may play an important role in IL-1-mediated cartilage breakdown and inflammation in arthritis.^21,22,24,27^ In addition, PPARγ cartilage-specific knockout (KO) mice exhibit spontaneous OA, and mice with inducible cartilage-specific PPARγ KO, subjected to the DMM model of OA, experienced more rapid development of OA than control mice.^43,44^

GPR55 is activated by a number of exogenous and endogenous cannabinoids.^14,15,45^ GPR55 is expressed in both normal and OA human chondrocytes at the protein level,^19^ and abnormal cannabinoid O-1602 was shown to reduce inflammatory pain in a rat model of arthritis, which was thought to be mediated by GPR55.^46^ McHugh et al.^16^ reported that phytocannabinoid Δ^9^-tetrahydrocannabinol and endogenous cannabinoid AEA are ligands of GPR18, suggesting that GPR18 may also act as a cannabinoid receptor; however, GPR18 is yet to be identified in articular cartilage.^16^

Cannabinoid receptor TRPV1 is expressed by human OA chondrocytes and human OA and RA synovial fibroblasts.^29,32^ TRPV1 acts as a receptor for the endogenous cannabinoid AEA and has also been shown to bind the phytocannabinoid: cannabidiol (CBD).^17,18^ CBD has anti-inflammatory and hypoalgesic effects in a rat model of acute inflammation, through TRPV1 activation.^47,48^

Although previous studies have shown that cannabinoids display chondroprotective properties, the expression of cannabinoid receptors within OA cartilage is poorly defined. This study aimed to identify and quantify the expression levels of CB1, CB2, GPR55, GPR18, TRPV1, PPARα, and PPARγ in OA cartilage and the underlying bone. Subchondral bone was investigated to determine whether potential chondroprotective effects of cannabinoids may be mediated by receptors on osteocytes and/or chondrocytes. Cannabinoids have been demonstrated to have effects on bone metabolism.^49^ Immunohistochemistry was used to determine receptor localization and whether expression levels altered during degeneration.

## Materials and Methods

### Articular cartilage tissue

Articular cartilage (Table 1) was obtained under the ethics committee approval held by the Sheffield Musculoskeletal Biobank (STH1606, SMB002). All patients provided written, informed consent before participation. Cartilage blocks were taken from waste tissue within each anatomic compartment of the knee (medial and lateral tibio-femoral and patello-femoral compartments) during total knee replacement for OA.

**Table 1.** **Human Cartilage Samples, Macroscopic and Microscopic Grades of Degeneration**

Sample	Age	Macroscopic grade	Microscopic grade
1	82	0	2.5
2	60	0	3
3	65	1	3
4	72	0	3.5
5	72	2–3	4
6	67	3	4
7	73	0	5
8	72	2	5
9	57	0	5
10	72	1	5
11	73	3	8
12	81	1	8.5
13	72	2	8.5
14	65	3	9
15	57	1	9
16	57	0	9.5
17	83	2	10
18	82	2	10
19	67	1	11
20	67	3	13.5
21	81	3	13.5
22	89	2	14.5
23	57	3	14.5
24	72	2	14.5
25	72	3	14.5
26	79	4	15
27	57	2	16
28	83	2	16.5
29	60	3	17.5
30	57	2	17.5
31	73	4	19.5
32	72	4	19.5
33	74	3	19.5
34	67	4	20.5
35	72	4	21
36	74	3	21.5
37	82	4	21.5
38	72	4	22

### Histology

**FIG. 1.**
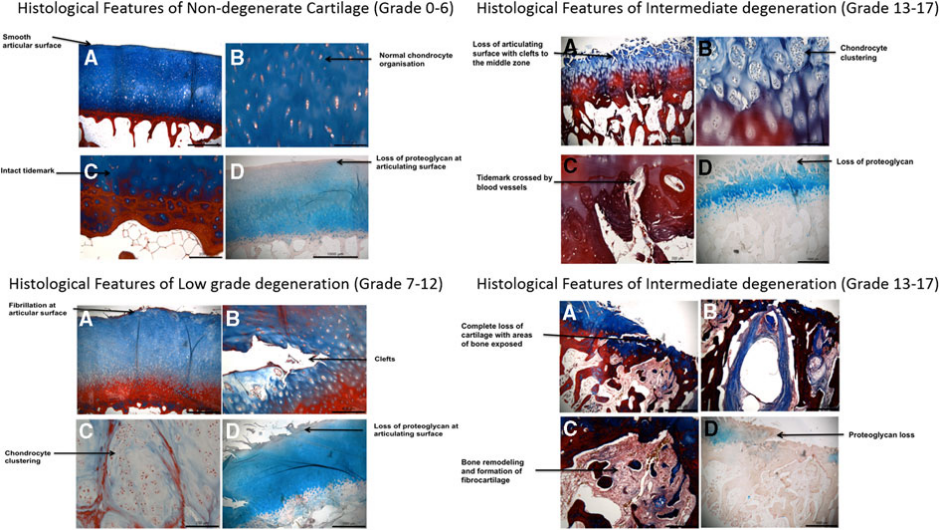
Histological features used to inform grading of degeneration. Representative images shown to highlight key features seen in the different grades of degeneration, which link to the histological grading system used (Table 2). **A–D** demonstrate key features of each grade of degeneration.

Cartilage tissue was fixed in 10% v/v formalin in phosphate-buffered saline (Sigma-Aldrich) followed by decalcification (Leica). Graded solutions of industrial methylated spirits (IMS; Fisher) were used to dehydrate samples followed by clearing in Sub-X (Leica). Samples were embedded in paraffin wax and 4 μm sections were mounted on slides. Sections were dewaxed in Sub-X, rehydrated in IMS, washed in distilled water, stained in 1% w/v Alcian blue/glacial acetic acid (pH 2.4) for 15 min, counter stained in 1% w/v aqueous neutral red for 1 min, or stained with Masson trichrome (Leica) according to the manufacturer's instructions. Sections were dehydrated and mounted. Cartilage tissue was graded using the Mankin scores (0–14) with additional scores for abnormal features (0–4) and cartilage thickness (0–4) being based on the OARSI scoring system (Fig. 1 and Table 2).^50,51^ The total scores were used to determine the overall grade of the cartilage as nondegenerate (scores 0–6), low grade degenerate (scores 7–12), intermediate degenerate (scores 13–17), and severe degenerate (scores 18–22; Table 1).

**Table 2.** **Histological Grading of Cartilage Sections**

Item	Classification	Score
Structure (H&E/MT)	Normal	0
	Superficial fibrillation surface irregularities	1
	Pannus and surface irregularities	2
	Clefts to transitional zone	3
	Clefts to radial zone	4
	Clefts to calcified zone	5
	Complete disorganization	6
Cells (H&E/MT)	Normal	0
	Diffuse hypercellularity (<25%)	1
	Clusters (25–75%)	2
	Hypocellularity (>75%)	3
Proteoglycan content (Alcian Blue)	Normal	0
	Slight reduction (surface zone loss)	1
	Moderate reduction (upper 1/3 loss)	2
	Severe reduction (into upper 2/3 deep zone)	3
	No staining present or very limited in bottom 1/3	4
Tidemark integrity (H&E/MT)	Intact	0
	Crossed by blood vessels	1
Abnormal features (H&E/MT)	None	0
	Denudation: surface sclerotic bone or reparative tissue including fibrocartilage microfractures with limited repair to bone surface	2
	Deformation: bone remodeling (more than osteophyte formation) includes microfracture with fibrocartilaginous and osseous repair extending above previous surface	4
Cartilage thickness (H&E/MT)	Normal smooth articulating surface	0
	Thinning of superficial zone	1
	Thinning into middle zone (>25% surface area)	2
	Thinning into deep zone (>25% surface area)	3
	Areas where bone exposed (>25% surface area)	4

H&E, hematoxylin and eosin; MT, Masson trichrome.

### Immunohistochemistry

Immunohistochemistry was used to detect the expression and localization of cannabinoid receptors present in 38 articular cartilage samples, representing a range of degeneration stages (Table 1). Tissue sections were dewaxed in Sub-X (Leica) and rehydrated in IMS; before endogenous peroxide blockade, enzymatic antigen retrieval was performed for 30 min at 37°C in 0.01% w/v chymotrypsin (Sigma), 0.1% w/v CaCl_2_ in Tris buffered saline (TBS), and samples were blocked in 25% v/v goat serum/1% w/v BSA in TBS for 1 h at room temperature (Abcam). Samples were incubated overnight at 4°C with rabbit polyclonal antibodies (Abcam) against CB1 (1:100) (ab23703), CB2 (1:50)(ab3561), PPARα (1:250)(ab8934), PPARγ (1:50)(ab19481), and TRPV1 (1:1000)(ab63083), and rabbit polyclonal antibodies from Acris (Acris GmbH) against GPR55 (1:100)(SP4239P) and GRP18 (1:200)(AP06930PU). IgG controls (abcam) were performed for each primary antibody at equivalent IgG concentrations. Samples were washed in TBS and incubated with biotinylated goat antirabbit secondary antibody (1:300, Abcam) for 30 min before detection by the formation of horseradish peroxidase-streptavidin-biotin complexes (Vector Laboratories) with 3,3′-diaminobenzidine tetrahydrochloride as a substrate (Sigma-Aldrich). Samples were counterstained in hematoxylin (Leica Microsystems), dehydrated, and mounted in pertex before microscopy.

### Microscopic analysis

All slides were examined with an Olympus BX51 microscope and images were captured by a digital camera and Capture Pro OEM v8.0 software (Media Cybernetics). Histological sections were analyzed, features noted, and images were captured to document their histological appearance. Grading was performed by two independent researchers (S.L.D. and C.L.L.M.) and the grades were averaged. For immunochemical analysis of tissue sections, a total of 200 chondrocytes were counted in each section in each zone of the cartilage where present (superficial, middle, and deep zone, clusters and osteocytes; please note degenerate cartilage does not have surface zones) and determined whether immunopositive or immunonegative. The number of immunopositive cells was expressed as a percentage of the total count within each region. Patient samples were grouped based on histological grades (nondegenerate [scores 0–6], low grade degenerate [scores 7–12], intermediate degenerate [scores 13–17], and severe degenerate [scores 18–22]; Table 1).

### Statistical analysis

The percentage of immunopositive cells for cannabinoid receptors was compared between each cartilage zone using the nonparametric Kruskall–Wallis with a Conover–Inman *post hoc* test. Nonparametric linear regression was used to determine the relationship between the grade of degeneration compared to immunopositivity within each of the cartilage zones. All statistical analyses were performed using StatsDirect.

## Results

### Cannabinoid receptor expression in OA cartilage and bone

**FIG. 2.**
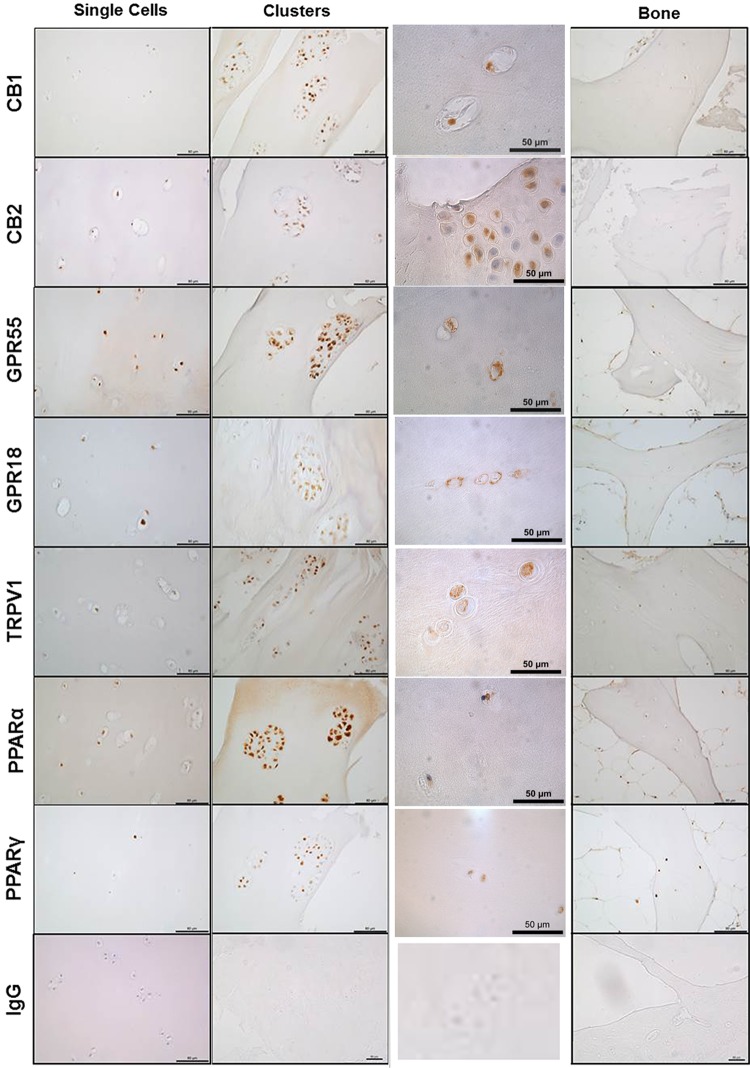
Immunohistochemical staining for cannabinoid receptor expression, CB1, CB2, GPR55, GPR18, TRPV1, PPARα, and PPARγ in OA articular cartilage and bone together with rabbit IgG control. CB, cannabinoid; GPR, G-protein-coupled receptor; OA, osteoarthritis; PPAR, peroxisome proliferator-activated receptors; TRPV, transient receptor potential vanilloid.

**FIG. 3.**
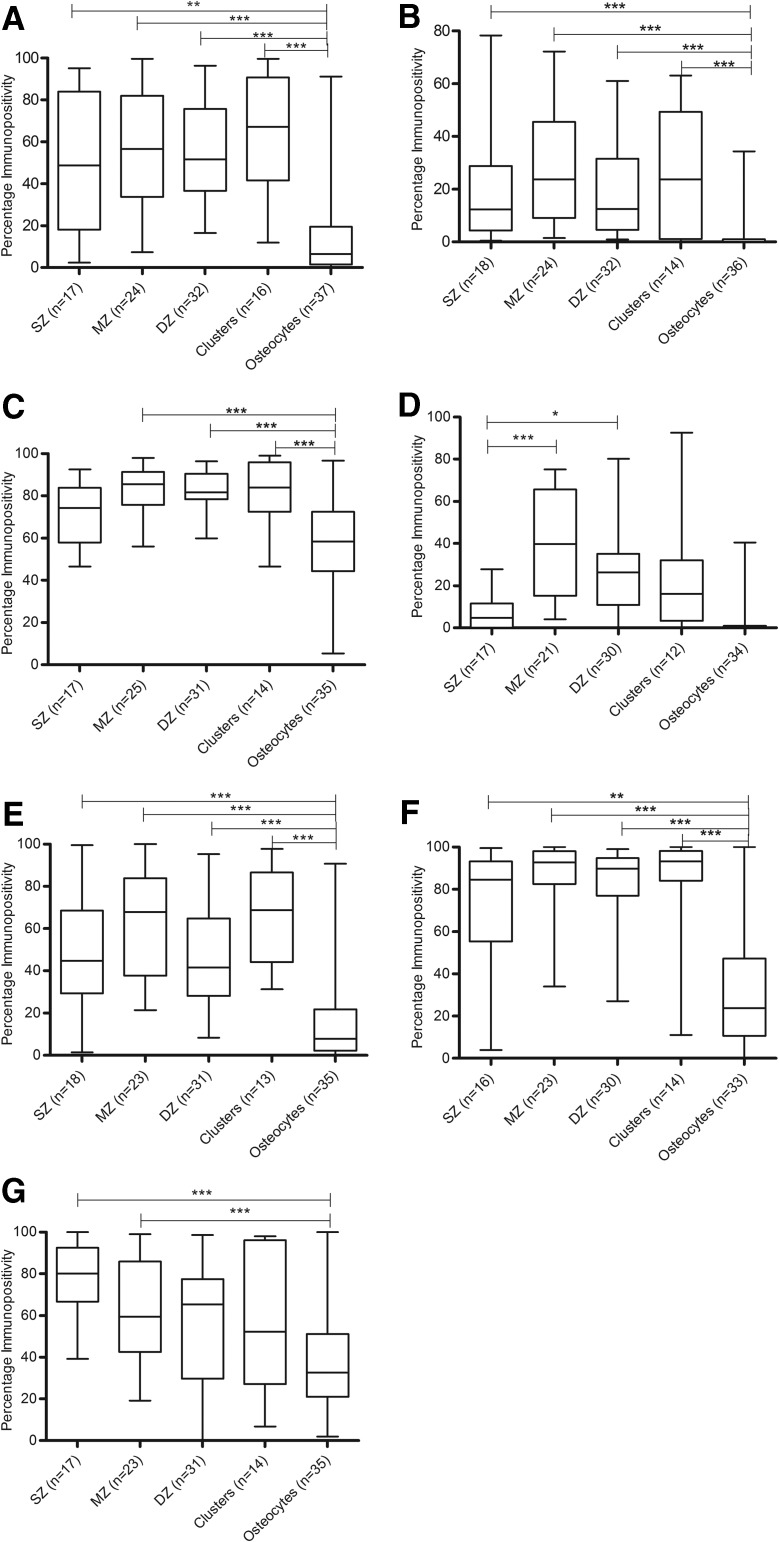
Quantitative analysis of immunopositivity of cannabinoid receptors in each zone of articular cartilage and bone. **(A)** CB1 immunopositivity. **(B)** CB2 immunopositivity. **(C)** GPR55 immunopositivity. **(D)** GPR18 immunopositivity. **(E)** TRPV1 immunopositivity. **(F)** PPARα immunopositivity. **(G)** PPARγ immunopositivity. *p<0.05, **p<0.01, ***p<0.001. DZ, deep zone; MZ, middle zone; SZ, superficial zone.

CB1, CB2, GPR55, GPR18, TRPV1, PPARα, and PPARγ immunopositivity was observed in single chondrocytes, chondrocyte clusters, and in osteocytes in the underlying bone (Fig. 2). CB1, CB2, TRPV1, and PPARα expression levels were significantly higher in the chondrocytes present in the superficial zone, middle zone, deep zone, and clusters than staining in osteocytes (all *p* values are less than 0.01, see figure for specific *p* values; Fig. 3A, B, E and F). GPR55 expression was significantly higher in the middle zone, deep zone, and clusters than staining in the osteocytes (*p*<0.001; Fig. 3C). Increased expression of GPR18 was detected in the middle (*p*<0.001) and deep zone (*p*<0.05) compared to the superficial zone of the cartilage (Fig. 3D). Expression of PPARγ was significantly higher in the superficial and middle zones than in the osteocytes (*p*<0.001; Fig. 3G).

**FIG. 4.**
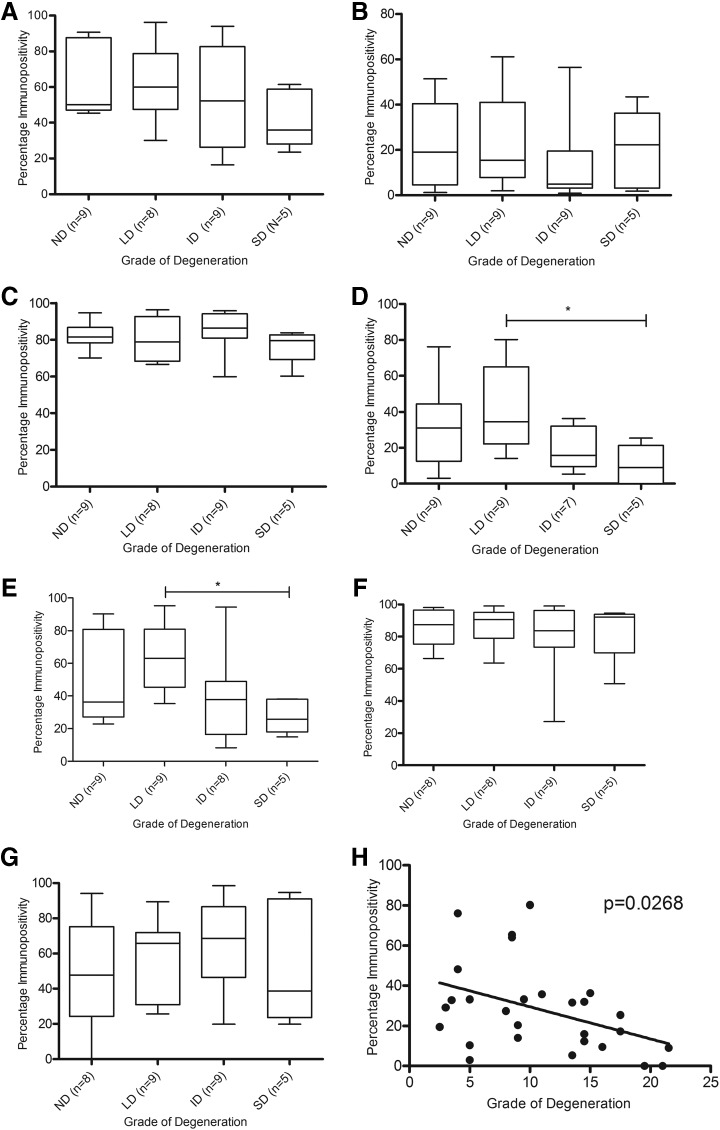
Quantitative analysis of immunopositivity of cannabinoid receptors in the deep zone of OA articular cartilage. **(A)** CB1 immunopositivity. **(B)** CB2 immunopositivity. **(C)** GPR55 immunopositivity. **(D)** GPR18 immunopositivity. **(E)** TRPV1 immunopositivity. **(F)** PPARα immunopositivity. **(G)** PPARγ immunopositivity. **(H)** Correlation of grade of cartilage degeneration and GPR18 immunopositivity. *p<0.05. ID, intermediate degeneration; LD, low degeneration; ND, nondegenerate; SD, severe degeneration.

There was no significant difference between CB1, CB2, GPR55, GPR18, TRPV1, PPARα, and PPARγ expression in chondrocytes in the superficial zone, middle zone, or those present in clusters between grades of degeneration (data not shown). There was no significant difference in CB1, CB2, GPR55, PPARα, and PPARγ expression between grades of degeneration in the deep zone of the cartilage (Fig. 4A–C, F and G); however, a significant decrease in GPR18 and TRPV1 expression in the severe degenerate samples compared to the low degenerate samples within the deep zone of the cartilage was seen (*p*<0.05; Fig. 4D,E). Regression analysis of GPR18 immunopositivity and the histological grade of degeneration confirmed that there was a significant negative correlation between GPR18 expression and grade of degeneration in the chondrocytes in the deep zone of the cartilage (*p*=0.0268; Fig. 4H), which was not the case for TRPV1 (*p*>0.05; data not shown).

**FIG. 5.**
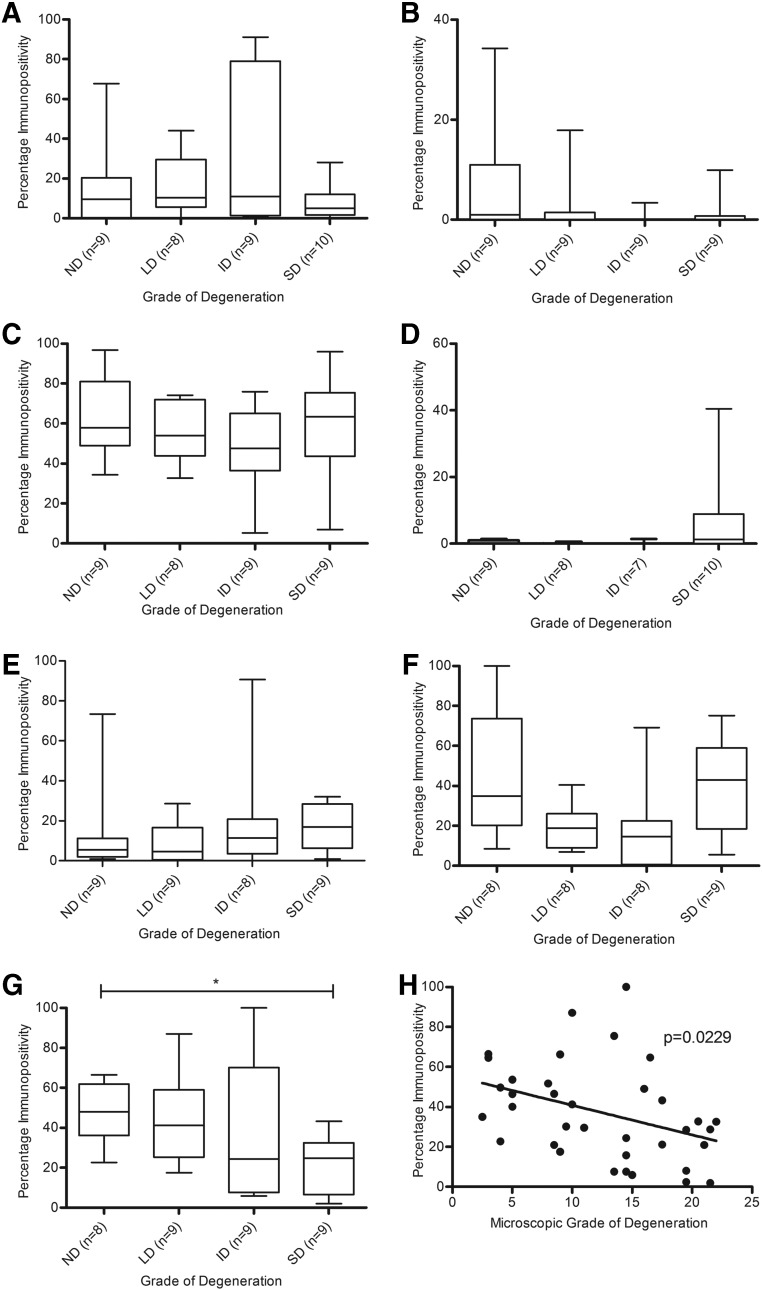
Quantitative analysis of immunopositivity of cannabinoid receptors in osteocytes. **(A)** CB1 immunopositivity. **(B)** CB2 immunopositivity. **(C)** GPR55 immunopositivity. **(D)** GPR18 immunopositivity. **(E)** TRPV1 immunopositivity. **(F)** PPARα immunopositivity. **(G)** PPARγ immunopositivity. **(H)** Immunopositivity within osteocytes for PPARγ within the underlying bone correlation with grade of cartilage degradation for tissue sample. *p<0.05.

Osteocyte immunopositivity for CB1, CB2, GPR55, GPR18, TRPV1, and PPARα was not affected by grade of degeneration (Fig. 5A–F). However, PPARγ expression was significantly decreased in the osteocytes in severe degenerate samples compared to nondegenerate samples (*p*<0.05; Fig. 5G), with an inverse correlation observed between PPARγ immunopositivity and grade of degeneration (*p*=0.0229; Fig. 5H).

## Discussion

This study demonstrated that both chondrocytes and osteocytes in the underlying bone express CB1, CB2, GPR55, GPR18, TRPV1, PPARα, and PPARγ receptors, demonstrating that these cells have the potential to respond to endocannabinoids expressed within the joint and also synthetic cannabinoids. These receptors may be potential targets for arthritis therapies.^52^ The expression of the majority of the receptors was similar across all zones of cartilage and within single cells to the same extent as clusters. However, higher receptor expression was seen in chondrocytes than in osteocytes, suggesting that chondrocytes may be more responsive to cannabinoids than to osteocytes; therefore, chondroprotective effects of cannabinoids are more likely to be mediated by chondrocytes than by osteocytes. In relation to this, CB2 receptors appeared to have minimal effects on bone in DMM-induced OA as CB2-deficient mice showed little or no changes in subchondral bone.^8^ To date, few studies have investigated the expression of cannabinoid receptors within the joint and none have investigated expression across the zones of cartilage, an important finding as the surface zones are lost during degeneration and thus may impact on the responsiveness of cells.

Other workers have demonstrated expression of CB1 and CB2 by bovine articular chondrocytes,^20^ synovia of patients with OA and RA,^30^ and human chondrocytes and fibroblast-like synoviocytes.^19,39^ Here, we demonstrate CB1 and CB2 localization within the cell membrane and cytoplasm of chondrocytes in all zones of cartilage and in the underlying osteocytes. Although CB1 and CB2 are cell membrane receptors, they have been shown to redistribute and traffic to different cellular components following ligand binding.^53^ In mice, deficiency of CB2 receptors has been associated with more rapid development of OA during aging and in mice with DMM-induced OA, suggesting the importance of this receptor in chondroprotection.^8^

GPR55 has previously been shown to be expressed by both normal and OA chondrocytes,^19^ which agrees with the findings of this study, where we also demonstrated no significant difference between grade of degeneration and GPR55 expression. GPR18 is primarily expressed in testes and spleen and in addition is expressed in other tissues and cells involved in endocrine and immune functions.^54^ We have shown, for the first time, that GPR18 is expressed by chondrocytes in OA cartilage. Immunopositivity for GRP18 was highest within the middle and deep zone than in the superficial zone of the cartilage, although the percentage of cells with immunopositivity decreased in the deep zone of the cartilage with increasing grade of degeneration. These findings indicate that GPR18 may play a role in the pathogenesis of OA and may have different functions in the middle and deep zones of the cartilage, as chondrocytes in different zones are known to express different molecules and display distinct functions.^2^ This novel finding of GPR18 in cartilage and bone suggests a role in cartilage and bone metabolism, which requires further investigation.

TRPV1 acts as an endogenous cannabinoid receptor for AEA and has also been shown to bind phytocannabinoids including CBD.^17,18^ TRPV1 is mainly expressed by nociceptive neurons and is activated by noxious heat and capsaicin.^55,56^ TRPV1 expression has been associated with arthritic pain in animal models and is increased in knee innervation in a rat OA model compared to control animals.^57^ TRPV1 KO mice have reduced thermal hyperalgesic sensitivity in an adjuvant-induced arthritis model.^58^ In humans, TRPV1 has been shown to be expressed in OA chondrocytes at the mRNA level and OA and RA synovial fibroblasts at the mRNA and protein levels.^29,32^ Any effects of chondrocyte TRPV1 stimulation on pain may be indirect. However, it may be postulated that an increase in TRPV1 expression would be associated with OA disease progression. Interestingly, in this study, it was shown that TRPV1 expression was significantly decreased in the deep zone of the cartilage with severe degeneration compared with cartilage with low degeneration. However, a decrease in TRPV1 expression has been associated with a differentiated phenotype in human OA chondrocytes cultures,^29^ suggesting that a decrease in TRPV1 expression observed in OA cartilage may be due to more differentiated chondrocytes in the deep zone in severe degeneration during OA.^59^

PPARγ immunopositivity increased in chondrocytes in the superficial zone of cartilage, although this failed to reach significance and immunopositivity was predominantly nuclear. In agreement with this finding, PPARγ was previously shown to be mainly expressed in the superficial zone of human OA cartilage and PPARγ expression has been shown to be nuclear in rat cartilage.^22,23^ In animal models of arthritis, the PPARγ agonist pioglitazone reduced the development and severity of cartilage lesions^60,61^ and the importance of PPARγ in cartilage development and homeostasis has recently been investigated,^44,62^ where PPARγ cartilage-specific KO mice exhibited a spontaneous OA phenotype.^44^ Mice displayed histological features of OA, including cartilage degradation, increased proteoglycan loss, hypocellularity, calcified cartilage, fibrillation, synovial inflammation, and fibrosis compared with age-matched controls.^44^

Similarly, in mice with inducible, cartilage-specific PPARγ KO with DMM-induced OA, OA development was accelerated. In addition, increased expression of catabolic and inflammatory markers was observed in chondrocytes.^44^ We have shown that there was no significant difference in PPARγ expression in OA cartilage compared to grade of degeneration; however, PPARγ was decreased in osteocytes in the underlying bone of OA cartilage with increasing grade of degeneration. Cartilage-specific PPARγ-deficient mice displayed bone defects, including reduced length of long bones, bone density, and trabecular bone thickness,^62^ suggesting that PPARγ is involved in bone metabolism. PPARγ agonists rosiglitazone and pioglitazone reduced bone erosions and inflammatory bone loss in a collagen-induced arthritis model,^63^ and PPARγ signaling pathway genes are upregulated during the osteoblast mineralization process.^64^ Although reduced expression of PPARγ is thought to play a role in the pathogenesis of OA,^22,25,44^ its role in bone metabolism during OA remains to be defined.

There was no significant difference in PPARα expression observed between different microscopic grades of OA cartilage. In agreement with our study, Afif et al.^22^ showed that PPARα was expressed by human articular chondrocytes and no differences in expression were observed in different grades of OA.^22^ *In vivo* studies have also demonstrated that PPARα expression levels did not change during the progression of OA in animal models.^25^

We have previously shown that synthetic cannabinoid WIN-55 reduces the gene expression of MMP-3 and MMP-13 in OA chondrocytes;^9^ however, the mechanisms by which this occurs are unknown. Here, we show that in chondrocyte cultures multiple cannabinoid receptors are expressed, suggesting that WIN-55 may mediate its effects through one or more of these receptors.

## Conclusions

This study demonstrates that chondrocytes within OA cartilage and osteocytes within the underlying bone express a wide range of cannabinoid receptors. However, cannabinoid receptor expression of CB1, CB2, GPR55, PPARα, and PPARγ in cartilage does not appear to be associated with grade of degeneration. Changes in TRPV1 and GPR18 expression were seen in the deep zone of the cartilage with increasing grade of degeneration, although whether these receptors are important in OA disease mechanisms remains to be defined. The maintenance of cannabinoid receptor expression shown in this study during OA suggests that these cells would remain responsive to cannabinoid receptor-targeted therapy using selective ligands. It is important to further identify the role of these receptors within normal and OA cartilage to elucidate their potential as possible targets in the treatment of OA.

## Abbreviations Used

AEA: arachidonoylethanolamide
CB: cannabinoid
CBD: Cannabidiol
DMM: destabilized medial meniscus
GPR: G-protein-coupled receptor
IL: interleukin
IMS: industrial methylated spirits
KO: knockout
MMPs: matrix metalloproteinases
OA: osteoarthritis
PPAR: peroxisome proliferator-activated receptors
RA: rheumatoid arthritis
TRPV: transient receptor potential vanilloid
TBS: Tris buffered saline
WIN-55: WIN 55, 212-2 mesylate
WT: wild type
